# Rabies Virus Infection Induces Type I Interferon Production in an IPS-1 Dependent Manner While Dendritic Cell Activation Relies on IFNAR Signaling

**DOI:** 10.1371/journal.ppat.1001016

**Published:** 2010-07-22

**Authors:** Elizabeth J. Faul, Celestine N. Wanjalla, Mehul S. Suthar, Michael Gale, Christoph Wirblich, Matthias J. Schnell

**Affiliations:** 1 Department of Microbiology and Immunology, Jefferson Medical College, Thomas Jefferson University, Philadelphia, Pennsylvania, United States of America; 2 Jefferson Vaccine Center, Jefferson Medical College, Thomas Jefferson University, Philadelphia, Pennsylvania, United States of America; 3 Department of Immunology, University of Washington School of Medicine, Seattle, Washington, United States of America; Mount Sinai School of Medicine, United States of America

## Abstract

As with many viruses, rabies virus (RABV) infection induces type I interferon (IFN) production within the infected host cells. However, RABV has evolved mechanisms by which to inhibit IFN production in order to sustain infection. Here we show that RABV infection of dendritic cells (DC) induces potent type I IFN production and DC activation. Although DCs are infected by RABV, the viral replication is highly suppressed in DCs, rendering the infection non-productive. We exploited this finding in bone marrow derived DCs (BMDC) in order to differentiate which pattern recognition receptor(s) (PRR) is responsible for inducing type I IFN following infection with RABV. Our results indicate that BMDC activation and type I IFN production following a RABV infection is independent of TLR signaling. However, IPS-1 is essential for both BMDC activation and IFN production. Interestingly, we see that the BMDC activation is primarily due to signaling through the IFNAR and only marginally induced by the initial infection. To further identify the receptor recognizing RABV infection, we next analyzed BMDC from Mda-5−/− and RIG-I−/− mice. In the absence of either receptor, there is a significant decrease in BMDC activation at 12h post infection. However, only RIG-I−/− cells exhibit a delay in type I IFN production. In order to determine the role that IPS-1 plays *in vivo*, we infected mice with pathogenic RABV. We see that IPS-1−/− mice are more susceptible to infection than IPS-1+/+ mice and have a significantly increased incident of limb paralysis.

## Author Summary

Rabies virus (RABV) is a neurotropic RNA virus responsible for the deaths of the at least 40,000 to 70,000 individuals globally each year. However, the innate immune response induced by both wildtype and vaccine strains of RABV is not well understood. In this study, we assessed the pattern recognition receptors involved in the host immune response to RABV in bone marrow derived dendritic cells (DC). Our studies revealed that Toll like receptor (TLR) signaling is not required to induce innate responses to RABV. On the other hand, we see that IPS-1, the adaptor protein for RIG-I like receptor (RLR) signaling, is essential for induction of innate immune responses. Furthermore, we found that RIG-I and Mda-5, both RLRs, are able to induce DC activation and type I interferon production. This finding is significant as we can target unused pattern recognition receptors with recombinant RABV vaccine strains to elicit a varied, and potentially protective, immune response. Lastly, we show that IPS-1 plays an important role in mediating the pathogenicity of RABV and preventing RABV associated paralysis. Overall, this study illustrates that RLRs are essential for recognition of RABV infection and that the subsequent host cell signaling is required to prevent disease.

## Introduction

Type I interferon (IFN) was first identified as a “factor” that rendered cells resistant to viral infection [1]. It is now known that following viral infection, cells induce type I IFN, which in turn upregulates the expression of numerous antiviral proteins [2]. This class of cytokines is comprised of several genes including multiple IFN-α genes, a single IFN-ß gene, and the less well-defined IFN-ω, -ε, -τ, -δ, and -κ (for review [3]). In addition to having antiviral functions, type I IFNs play a part in activating the adaptive immune response following infection [4], [5], [6]. For instance, IFN-α/ß can strengthen the innate immune response by activating antigen presenting cells (APC). Additionally, following maturation in the presence of type I interferon and GM-CSF, monocyte-derived DCs more effectively stimulate an antigen-specific CD8^+^ T cell response than when differentiated with GM-CSF alone [7].

Viral infection can trigger the type I IFN response via various pattern recognition receptors (PRR), namely Toll-like receptors (TLR) and RIG-I-like receptors (RLR). In the case of negative stranded RNA viruses, the members of the TLR family that are generally involved in viral recognition, TLR-3 and TLR-7, are found on the endosomal membrane. To initiate the signaling cascade, TLR-3 binds double stranded RNA molecules [8], whereas TLR-7 recognizes immunomodulatory compounds (ie-imiquimod) [9] or single-stranded RNA molecules [10]. Although negative stranded RNA viruses do not produce double stranded RNA as part of their normal replication cycle, it is likely that abnormal replication products resulting from errors by viral RNA-dependent RNA polymerases give rise to some level of double stranded RNA in virus-infected cells [11]. TLR-3 and TLR-7 initiate signaling though different adaptor molecules, Trif and MyD88, respectively; however, the pathways converge on the phosphorylation of IRF-3. Following phosphorylation, IRF-3 forms protein dimers, which allow for its transport into the nucleus where it can bind to the IFN-ß promoter [12].

Alternatively, RNA viruses can be recognized in the cytoplasm by RLRs, namely RIG-I and Mda-5 [13]. These helicase-like proteins recognize double-stranded RNA and 5′ tri-phosphate groups [14]. In the case of rabies virus (RABV), the negative stranded RNA virus of interest in this study, the leader RNA remains unmodified [15], [16] and thus provides a potential ligand for these RLRs. Signaling by RIG-I and Mda-5 is mediated through the mitochondria-bound protein IPS-1, which is also referred to as MAVS, Cardif, or VISA [17], [18], [19], [20]. Similar to what is seen in TLR signaling, RLR signaling culminates with the activation and nuclear translocation of IRF-3 [19].

Rabies virus is a member of the Rhabdoviridae family. RABV has a relatively simple genome, comprised of just 5 proteins: the nucleoprotein, phosphoprotein (P), matrix protein, glycoprotein and the RNA dependent RNA polymerase. Infection with RABV can induce IFN-α/ß production rapidly *in vivo*. Furthermore, it was seen that a mouse's ability to induce type I IFN, as measured by serum concentrations 4 days post infection, positively correlates to the animal's resistance to RABV [21]. The type I IFN response is also important in driving immunity, as mice injected with anti-mouse IFN-α/ß antibody prior to infection with RABV were more sensitive to the virus than mice injected with a control antibody [22]. However, RABV has the ability to antagonize type I IFN induction [23]. Thus, shortly after infection of fibroblast cells, RABV-P prevents IRF-3 phosphorylation in order to suppress IFN-α/ß production [23].

Although IFN is induced after RABV infection, RABV is able to suppress the IFN response shortly after infection. Therefore, in order to study the receptors responsible for the initial induction of IFN, several groups have used recombinant viruses with lower levels of RABV-P. Using this method, it was determined that IFN-ß promoter activity was seen following recombinant RABV infection of VERO cells transfected with wildtype RIG-I, but not in cells transfected with dominant-negative mutant RIG-I [24], thus indicating a role for RIG-I in mounting an innate immune response to RABV. Additionally, following infection of human postmitotic neurons with RABV, Prehaud *et al.* saw an increased production of IFN-ß and TLR-3 mRNAs [25]. Furthermore, the expression of TLR-3 on cerebellar cortex tissues of individuals that had died of rabies, but not on an individual that died of cardiac arrest, verify the viral induced expression of TLR-3 in human brains *in vivo* [26]. This upregulation of TLR-3 following infection suggests a possible role for TLR-3 signaling in the innate recognition of RABV; however, TLR-3 activation needs to be further studied to conclusively define such a role. Although these results hint at the receptors responsible for interferon expression, there is no evidence that other PRR receptors, such as TLR-7 and Mda-5, do not also play a role. Furthermore, since the recombinant viruses used in some of these studies exhibit decreased pathogenicity, it is possible that a wildtype virus may act differently following infection.

In order to study the IFN-inducing pathways triggered by RABV, we needed to identify a cell type in which RABV-P is unable to antagonize type I IFN signaling. Of note, it has been seen that following infection of dendritic cells (DCs) with influenza, another negative stranded RNA virus, the DCs become infected, but this infection is non-productive [27]. Here, we sought to determine whether APCs were productively infected with RABV. Similar to previous reports that human DCs are susceptible to RABV infection [28], [29], we saw that mouse DCs became infected; however we also observed that very little viral progeny was released due to limited viral replication. Due to the overall suppression of viral transcription in RABV infected DCs there are presumably low levels of RABV-P that may not be able to inhibit interferon induction. Thus, we decided to utilize infection of DC to study the IFN-inducing capabilities of RABV and found that RLRs are responsible for viral recognition in DCs.

## Results

### RABV infection of antigen presenting cells results in type I IFN production

It has been previously shown that RABV-P can inhibit the phophorylation of IRF-3 in fibroblast cells [23], thus crippling the induction IFN-α/ß. However, RABV is able to infect a variety of cells including neurons [30] and antigen presenting cells (APC) [28], [29] in addition to fibroblasts. Thus, we wanted to determine whether RABV is able to inhibit IFN signaling in other cell types including DCs, which are known to induce the adaptive immune response.

**Figure 1:**
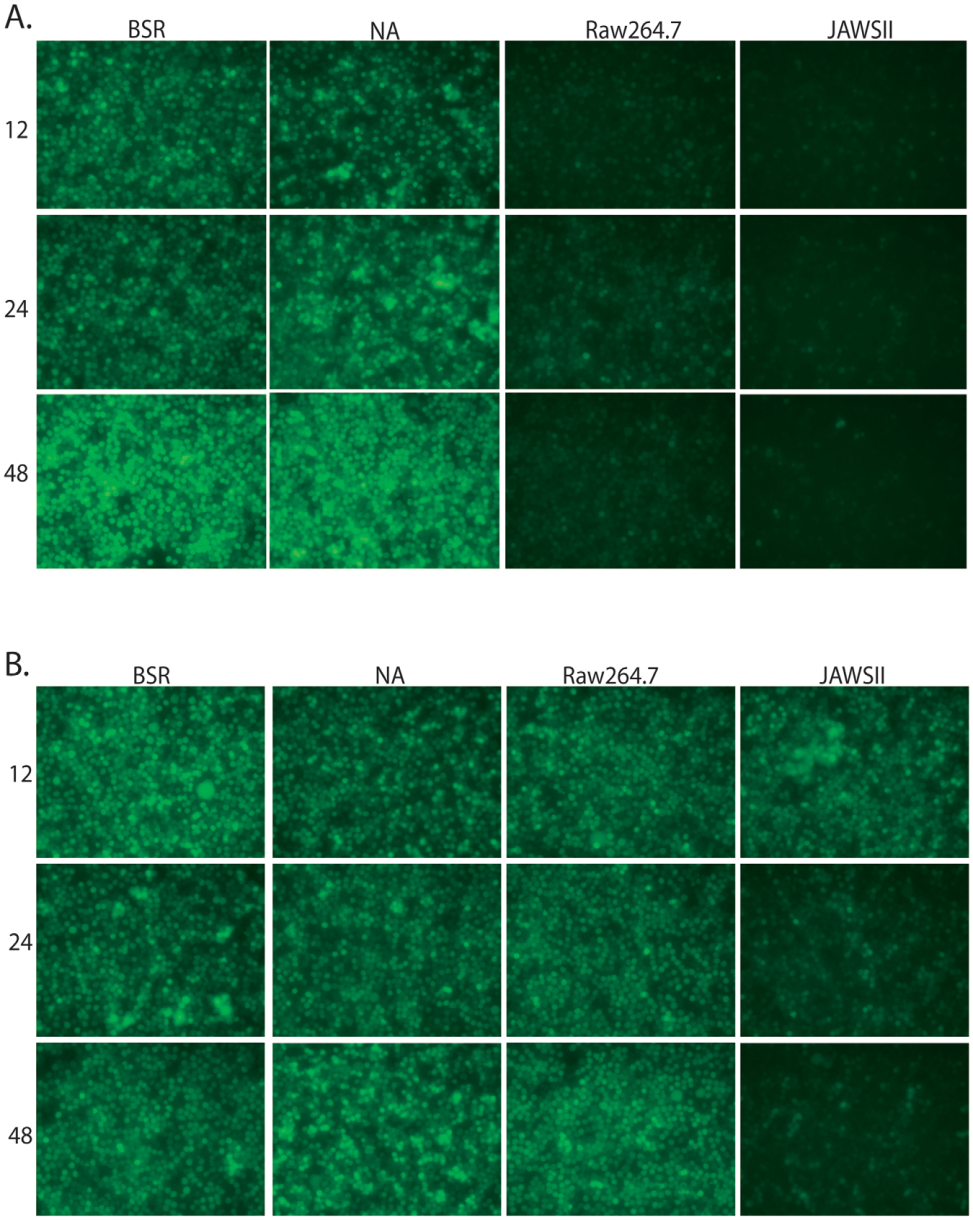
RABV infection of APCs, but not fibroblasts, induces type I IFN production. BSR, NA, Raw264.7, or JAWSII cells were infected with RABV (A) or UV-deactivated RABV (B) and the supernatant from the infected cells was UV-deactivated. Reporter cells were pre-treated with the UV-deactivated supernatant (diluted 1∶10) for 24h and then infected with VSV-GFP for 5h. Fluorescence indicates viral replication, and thus, lack of type I IFN. Photos are representative of two independent experiments.

In order to check for type I IFN production, we first infected a variety of cell types including fibroblasts (BSR), neuronal cells (NA), macrophages (Raw264.7) and DCs (JAWSII) with a RABV vaccine strain-based vector, SPBN. Following infection with RABV, cell supernatants were collected and subsequently UV-treated in order to deactivate any infectious virus but retain secreted cellular proteins, such as type I IFN. We then transferred the supernatants to reporter cells, which are sensitive to IFN. Twenty-four hours after supernatant transfer, reporter cells were infected with recombinant vesicular stomatitis virus expressing GFP (VSV-GFP, [31]) for 5–8h. VSV replication is highly sensitive to type I IFN [32], and thus, in the presence of type I IFN, the replication of VSV is suppressed [4]. Following infection with RABV, macrophages as well as DCs, but not fibroblasts or neuronal cells, produce type I IFN that inhibits VSV-GFP replication, as indicated by the lack of GFP expression (Figure 1A). Of note, when BSR, NA, Raw264.7, or JAWSII cells are originally treated with UV-deactivatecd RABV, the supernatants from these cells are unable to block VSV replication (Figure 1B); therefore, IFN is secreted only after RABV replication.

**Figure 2:**
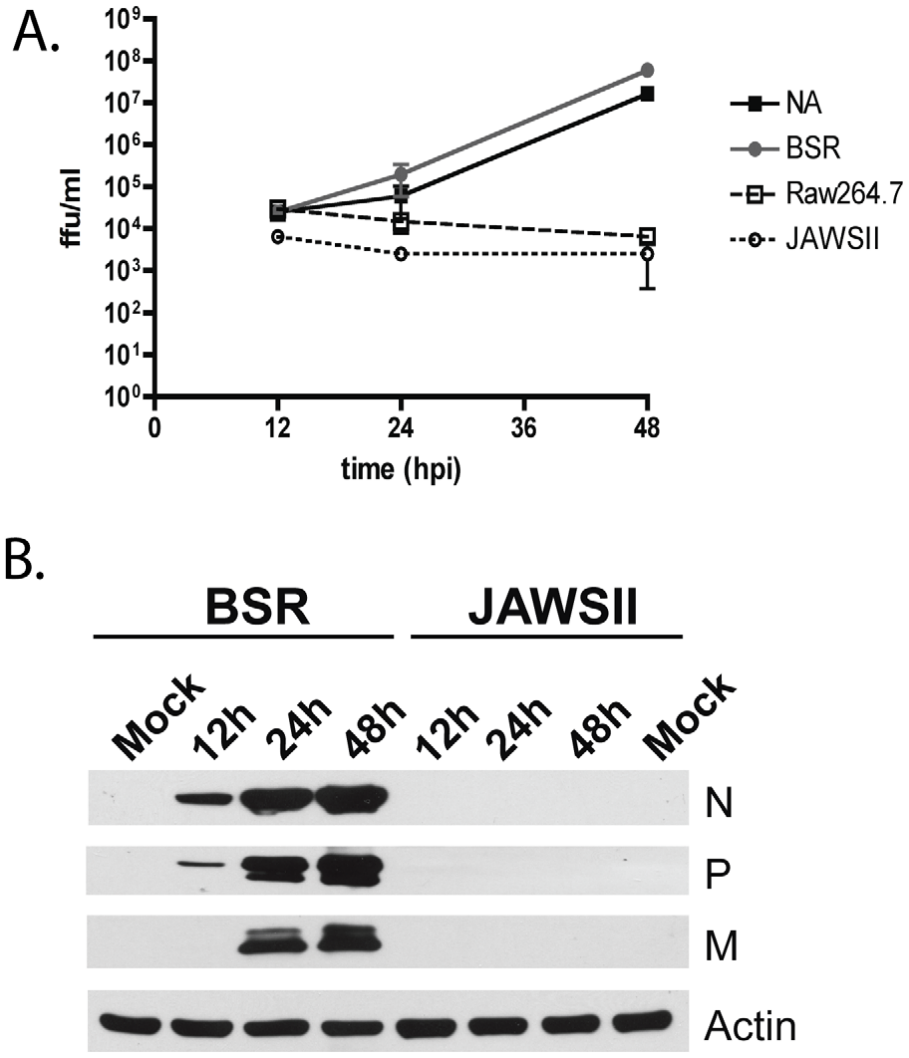
Infection of APCs is non-productive. (A) NA, BSR, Raw264.7, and JAWSII cells were infected with RABV at an MOI = 10. A one-step growth curve was generated by titering infectious RABV in the supernatant of the infected cells at various hours post infection as indicated. (B) Western blot analysis. BSR or JAWSII cells were infected with RABV (MOI of 10) and lysed 12, 24 or 48 h later. Proteins were separated by SDS-PAGE and subjected to Western blotting with mouse polyclonal antibodies specific for RABV (strain N2c), or a mouse monoclonal against actin (Actin). The RABV proteins N, P, and M are indicated.

In order to account for the increased amounts of type I IFN produced following RABV infection of macrophages and DCs when compared to the amount produced by fibroblast and neuronal cells, we did a one-step growth curve following infection of the various cell types. Supernatants from infected cells was titered on BSR cells, which are insensitive to type I IFN [4]. We detected that, although all four cell types were infected, only BSR and NA cells produce infectious virus (Figure 2A). There are two possible explanations for the defect in viral production observed here: either a block in viral replication or a defect in viral assembly. In order to compare viral transcription and replication in fibroblast and dendritic cell lines we used quantitative PCR. In fibroblast cells, we saw that the amount of RABV-N messenger RNA (mRNA) transcripts increased an average of 1.95 logs from 8 hours post infection (hpi) to 48 hpi. Similarly, the quantity of RABV-N genomic RNA transcripts (gRNA) increased an average of 1.2 logs from 8 hpi to 48 hpi. This data indicates that following infection of fibroblast cells both viral transcription (mRNA) and replication (gRNA) occurs. On the other hand, when looking at the quantity of RABV-N found in dendritic cells following infection we see that there was no increase in the number of mRNA or gRNA viral transcripts when comparing 8hpi to 48 hpi. Thus, it appears that RABV is able to enter APCs, but only limited viral transcription occurs following entry.

It is reasonable to assume that decreased levels of transcription might result in low levels of RABV-P. It has been previously shown that recombinant RABV expressing low amounts of RABV-P is unable to inhibit type I IFN induction [23]. Furthermore, we show by Western blotting that cell lysate from RABV infected JAWSII cells contained undetectable levels of RABV P 48hpi (Figure 2B). On the other hand, we were able to detect RABV P in lysates from infected BSR cells as early as 12 hpi after infection. This results support the conclusion that a very low level of RABV P within infected APCs is not able to block the induction of type I IFN and therefore is responsible for the increase in type I IFN production by these cells following infection.

**Figure 3:**
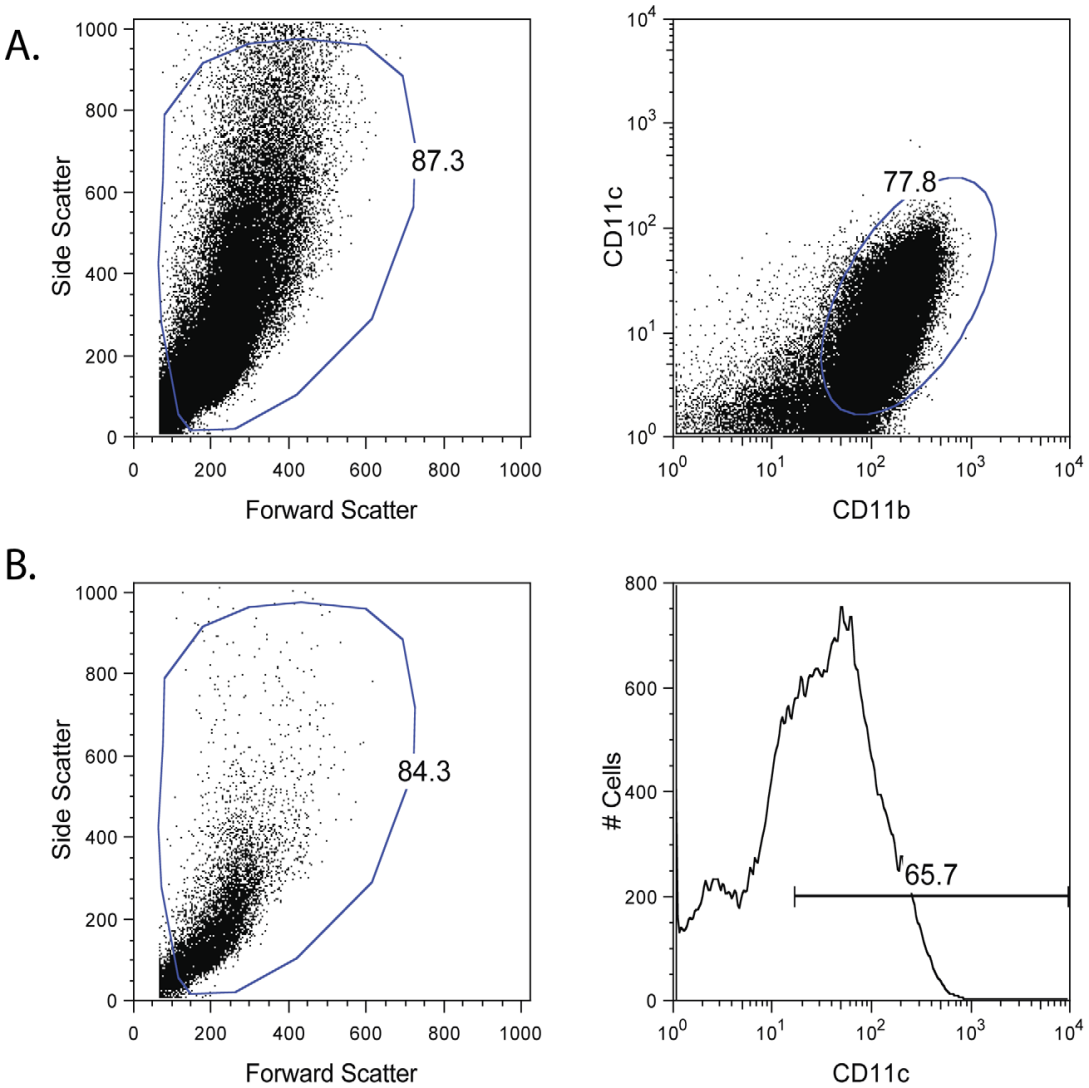
BMDC differentiation and gating. BMDC were derived from various mice by culture in media containing 10ng/ml GM-CSF for 7 days. (A) Following culture, the majority of viable cells (as determined by forward and side scatter) were CD11b+CD11c+. (B) When analyzing activation state or infection rates of BMDCs following stimulation the cells were first gated for viability (using forward and side scatter) and then gated for CD11c expression. Only CD11c+ cells were used in analysis. One representative Balb/c mouse is shown here, but this gating strategy was consistently used for all BMDC samples.

In order to better understand the interaction of RABV with host cells following infection, we sought to identify the pathway(s) responsible for type I IFN induction in infected cells. Since we detected that DCs make large amounts of IFN following RABV infection we decided to use bone marrow derived DCs (BMDC) in our studies. To differentiate BMDCs, we cultured the cells in the presence of 10 ng/ml GM-CSF. After 7 days the majority of cells have matured to DCs as shown by the expression of CD11b^+^CD11c^+^ (Figure 3). In order to identify the PRR that recognizes RABV, we isolated BMDC from mice deficient in various signaling components of PRR pathways. In each experiment, cells were stimulated, and the CD11c^+^ cell population (Figure 3) was analyzed for production of type I IFN and expression of CD86, a co-stimulatory molecule that is upregulated on activated DCs.

### Induction of type I IFN and DC activation following RABV infection is independent of TLR-3 and MyD88 signaling

**Figure 4:**
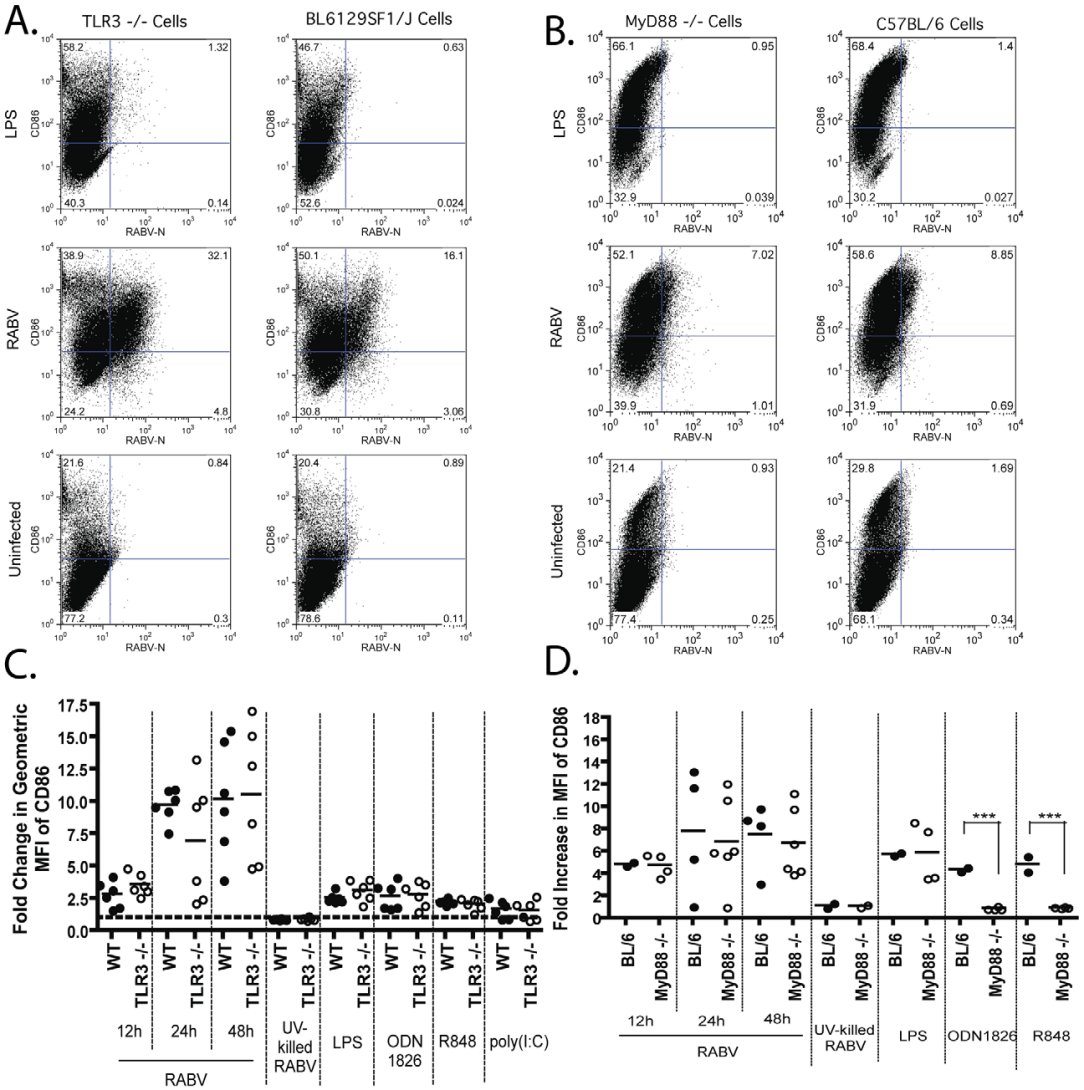
DC activation following a RABV infection occurs independent of TLR signaling. BMDC derived from TLR-3−/− mice (A and C) or MyD88−/− mice (B and D). (A–B) CD86 upregulation following RABV infection of BMDCs was monitored at 12, 24, and 48 hours post infection. One representative knock-out and wildtype mouse is shown at 12hpi. (C–D) For each sample the fold increase in activation was determined by dividing the geometric mean fluorescent intensity (MFI) of the sample by the MFI of the uninfected sample. (C) Data is from two independent experiments and each point is representative of BMDC from one mouse. (D) Data is from four independent experiments and each point is representative of BMDC from two pooled mice. Statistical significance was determined by T-test analysis and any significance is indicated, ***p<0.001.

First, we analyzed the role that TLR signaling plays in BMDC activation and type I IFN production following a RABV infection. It has been previously reported that following infection of human postmitotic neurons with RABV, there is an increased production of IFN-ß and TLR-3 mRNAs. In addition, treatment of neurons with poly(I:C), a TLR-3 agonist, generated a similar cytokine profile to that which was seen following RABV infection [25]. Thus, we differentiated BMDCs from TLR-3−/− and congenic wildtype mice and infected the cells with RABV. We then analyzed the infected cells for the presence of CD86 (Figure 4A). As shown in Figure 4C, there is no significant difference in the expression of CD86 on the cell surface of RABV infected BMDCs derived from TLR-3−/− or wildtype mice. As expected, TLR ligands that signal via other TLR receptors, namely TLR-4 (LPS), TLR-9 (ODN1826), and TLR-7/8 (R848), equally activate BMDCs derived from wildtype (wt) or TLR-3−/− mice. Interestingly, poly(I:C), a known ligand for TLR-3, was able to activate BMDC isolated from TLR-3−/− mice as well as wt mice. However, it has been previously shown that poly(I:C) can also signal through Mda-5 and that Mda-5 is the dominant receptor for mediating type I IFN induction following poly(I:C) stimulation in BMDCs [33], [34]. As the RLR pathway remains intact in TLR-3−/− mice, BMDC activation in TLR-3−/− cells following poly(I:C) stimulation is not inexplicable, but rather highlights the need for a better TLR-3 agonist. Taken as a whole and based on the fact that that RABV infection activated BMDC derived from both wt and TLR-3−/− mice equally, we conclude that TLR-3 signaling is not required for the activation of BMDCs following a RABV infection.

To our knowledge, TLR-7 has never been investigated in the context of a RABV infection and thus the role that it plays in type I IFN induction and DC activation following RABV infection is unknown. To analyze the function that TLR-7 has in the induction of type I IFN and DC activation, we isolated BMDCs from MyD88−/− and C57BL/6 mice. We detected an equal upregulation of CD86 on BMDCs from MyD88−/− and wildtype mice (Figure 4B). As expected, activation of MyD88−/− BMDCs is significantly reduced following stimulation with ODN1826 and R848, ligands for TLR-9 and TLR-7/8 respectively, both of which signal via MyD88 [35] (Figure 4D). Thus we conclude that, similar to TLR-3 signaling, the activation of BMDCs following a RABV infection occurs independently of MyD88 signaling.

In order to determine if TLR-3 and MyD88 signaling might have an impact on type I IFN production, supernatant from infected BMDCs was collected at various times post infection, and a VSV-sensitivity assay was performed. As seen with BMDC activation, both TLR-3 and MyD88 are dispensable in the induction of type I IFN (Table 1). We did not detect any VSV-GFP replication on reporter cells following pre-treatment with supernatant from TLR-3−/−, MyD88−/−, or wildtype BMDC, indicating the presence of type I IFN in the supernatant.

**Table 1:** Level of VSV-GFP replication on reporter cells following pre-treatment with UV-inactivated supernatants from RABV infected BMDC.

	SPBN, 12h	uninfected, 12h	UV-killed SPBN, 12h	SPBN, 24h	uninfected, 24h	SPBN, 48h	uninfected, 48h
**C57BL/6**	−	++	++	−	++	−	++
**MyD88−/−**	−	++	++	−	++	−	++
**BL6129SF1/J**	−	++	++	−	++	−	++
**TLR-3−/−**	−	++	++	−	++	−	++

−: Less than 1% of reporter cells are infected.

++: 100% of reporter cells are infected.

### Induction of type I IFN and DC activation following RABV infection requires the IPS-1 pathway

**Figure 5:**
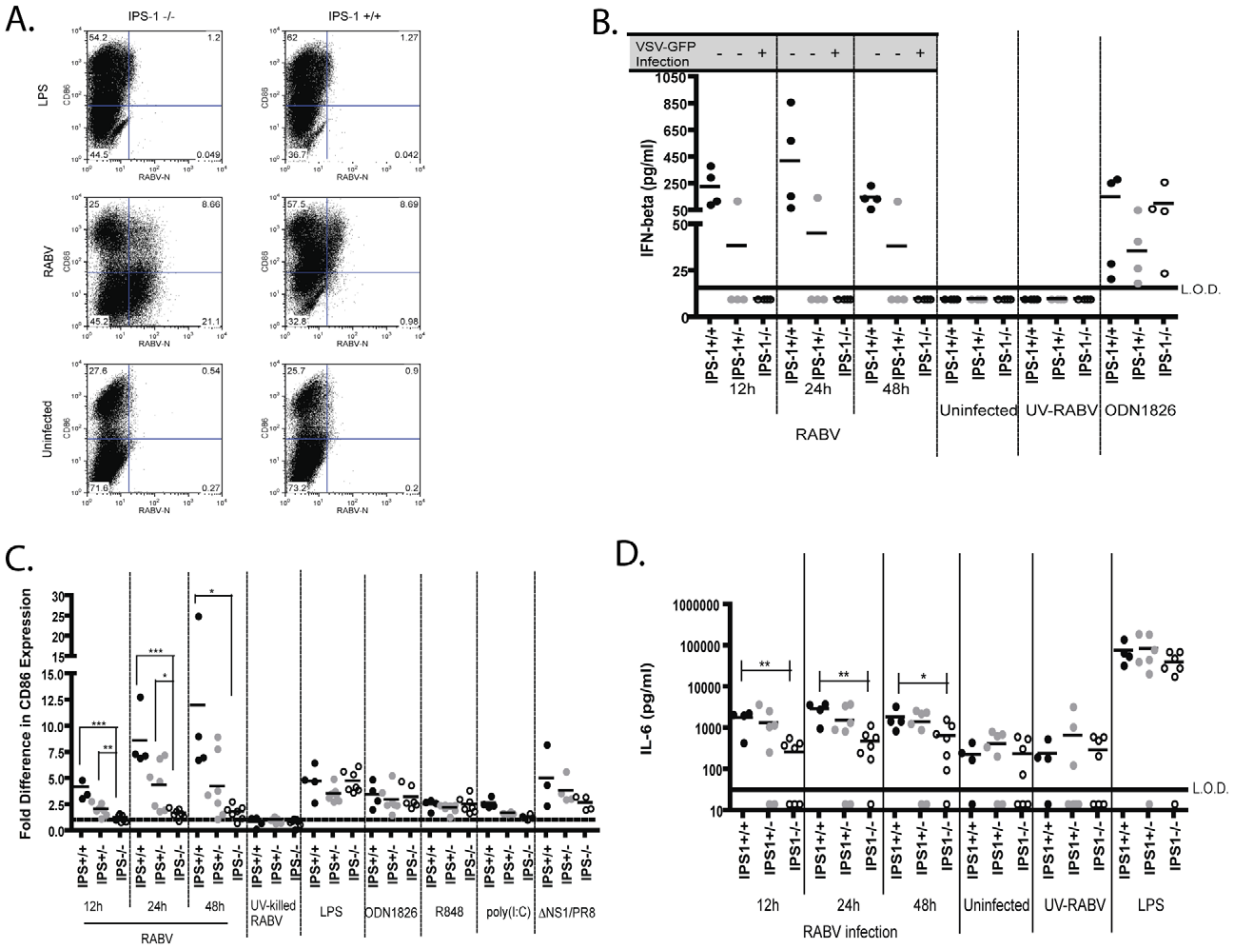
DC activation and IFN-production following RABV infection is dependent on IPS-1 signaling. CD86 expression on BMDCs following RABV infection was analyzed by flow cytometry. (A) One representative knock-out and wildtype mouse is shown 12hpi. (B) For each sample the fold increase in activation was determined by dividing the geometric mean fluorescent intensity (MFI) of the sample by the MFI of the uninfected sample. Data is from three independent experiments and each point is representative of BMDCs from one mouse. (C) IFN-α/ß levels were monitored by infectivity of VSV-GFP on reporter cells and an IFN-ß ELISA. The quantity of IFN-ß present in the supernatant of infected BMDCs is graphed here. Additionally, the infection (+) or lack of infection (−) by VSV-GFP following a 24h pretreatment with cell supernatant is indicated above the graph. Infection indicates viral replication, and thus, lack of type I IFN. (D) IL-6 levels were determined following infection of IPS-1 BMDCs by an ELISA. Statistical significance was determined by T-test analysis and any significance is indicated, *p<0.05, **p<0.01, ***p<0.001. L.O.D. is limit of detection.

Having excluded TLRs as the required receptors mediating BMDC activation and type I IFN production, we next looked at the potential role for RLR signaling. Hornung *et al.* showed that a recombinant RABV expressing low levels of RABV-P signals via RIG-I to induce IFN-ß promoter activity following infection. Furthermore, it was shown that the 5′-triphosphate on the leader sequence of RABV was the ligand for RIG-I [24]. To determine whether the RIG-I pathway is also activated in DCs following RABV infection, we isolated BMDCs from IPS-1+/+, +/−, or −/− mice. Our results indicate that following infection with RABV, IPS-1+/+ and IPS-1+/− BMDCs express high levels of CD86 on their surface (Figure 5A–B). Of note, IPS-1+/− BMDCs are slightly less activated then IPS-1+/+ cells. On the other hand, IPS-1−/− BMDCs express significantly lower levels of CD86 on their surface at all time points (Figure 5A–B). The TLR ligands LPS, ODN1826, and R848 equally activated all IPS-1 BMDC samples, indicating that the defect in the IPS-1 −/− BMDCs is specific to the RLR pathways (Figure 5B). As such, when cells are stimulated with RLR agonists, there is a defect in the activation of IPS-1−/− BMDCs when compared to IPS-1+/+ or +/− BMDCs. We see a low CD86 upregulation following both poly(I:C) stimulation and infection with a NS1-deficient strain of influenza (ΔNS1/PR8) (Figure 5B). It has been reported previously that poly(I:C) can signal via Mda-5 [33] and ΔNS1/PR8 signals exclusively via RIG-I [34]. Taken together this data indicates that BMDC activation is dependent on IPS-1 signaling following a RABV infection.

In order to determine whether type I IFN production by BMDC is also dependent on IPS-1 mediated signaling, we assayed for the presence of type I IFN in the supernatants of infected IPS-1 BMDCs by VSV-GFP sensitivity assays and quantified the amount of IFN-ß by ELISA. It was seen that supernatant obtained from IPS-1+/+ and IPS-1+/− BMDCs infected with RABV was able to inhibit VSV-GFP replication, and thus contained type I IFN. On the other hand, the VSV-GFP replication on reporter cells was not inhibited by pre-treatment with supernatants from RABV infected IPS-1−/− BMDCs (Figure 5C). Likewise, IPS-1 +/+ BMDCs produce on average 250 pg/ml IFN-ß while the IPS-1−/− BMDCs produced less than 16.7 pg/ml, if any, IFN-ß (Figure 5C). These results indicate that RABV infected IPS-1−/− BMDCs do not secrete type I IFN. Also consistent with the results seen for BMDC activation, IPS-1−/− cells stimulated with RLR agonists produced less type I IFN compared to IPS-1+/+ or +/− BMDCs (Table 2).

**Table 2:** Level of VSV-GFP replication on reporter cells following pre-treatment with UV-inactivated supernatants from TLR-agonist stimulated BMDC.

	LPS, 2ug/ml	ODN1826, 2uM	R848 1ug/ml	poly(I:C) 50ug/ml	ΔNS1/PR8
**C57BL/6**	−	−	+	ND	ND
**MyD88−/−**	++	++	++		
**BL6129SF1/J**	++	−	+	−	ND
**TLR-3−/−**	++	−	+	+	
**IPS-1+/+**	+	−	−	−	−
**IPS-1+/−**	+	−	−	−	−
**IPS-1−/−**	+	−	−	++	+
**C57BL/6**	−	−	+	−	ND
**Mda-5−/−**	−	−	+	+	
**RIG-I +/+**	+	−	+	−	ND
**RIG-I −/−**	+	−	+	−	

−: Less than 1% of reporter cells are infected.

+: Less than 50% of reporter cells are infected.

++: 100% of reporter cells are infected.

ND: not done.

It has been shown that IPS-1 mediated pathways are also capable of activating the NF-κB signaling cascade [36]. Thus, we quantified the amount of IL-6 in the supernatant of RABV infected BMDC isolated from IPS-1+/+, +/− and −/− mice (Figure 5D). We see that there is a significant decrease in IL-6 produced by IPS-1−/− BMDCs compared to IPS-1+/+ BMDCs. However, IPS-1−/− cells do secrete some IL-6 following infection with RABV, and thus, the use of IPS-1 independent pathways to induce NF-κB activation, in contrast to type I IFN activation, seems to be utilized.

### IPS-1 is the adaptor molecule for both Mda-5 and RIG-I

**Figure 6:**
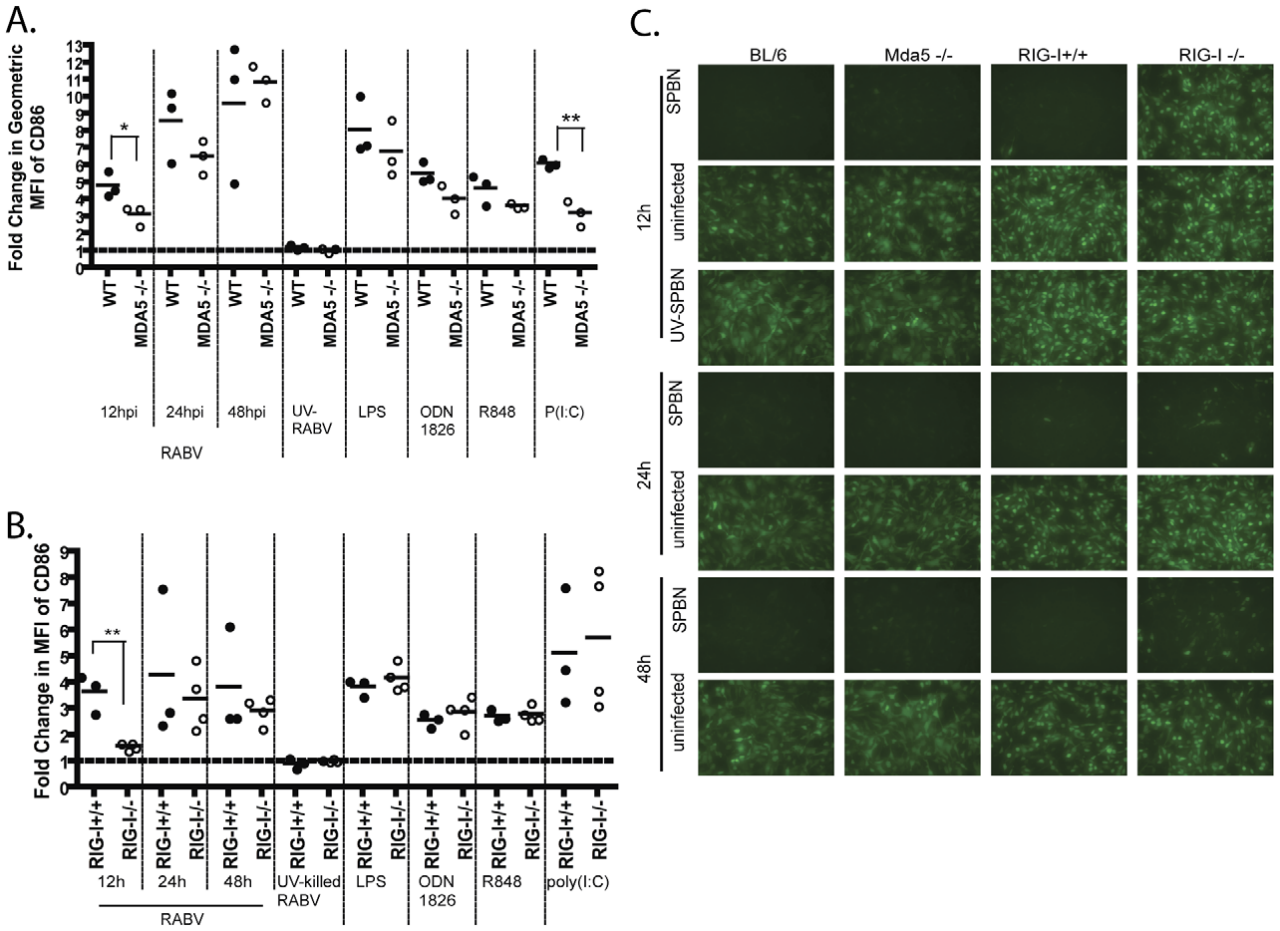
RABV infection triggers IFN production and DC activation via either Mda-5 or RIG-I. CD86 expression on BMDC following RABV infection was analyzed by flow cytometry 12, 24, and 48 h post infection. (A–B) For each sample the fold increase in activation was determined by dividing the geometric mean fluorescent intensity (MFI) of the sample by the MFI of the uninfected sample for Mda-5−/− (A) and RIG-I−/− (B). Each point in A and B is representative of BMDCs from one mouse and data for RIG-I is representative of two independent experiments. (C) IFN-α/ß levels were monitored by infectivity of VSV-GFP on reporter cells. Reporter cells were pre-treated with UV-inactivated BMDC supernatant from RABV infection. 24h after supernatant was applied, reporter cells were infected with VSV-GFP. Fluorescence indicates viral replication, and thus, lack of type I IFN. Statistical significance was determined by T-test analysis and any significance is indicated, *p<0.05, **p<0.01.

Mda-5 mediated induction of IFN-ß has been described to occur in response to plus-stranded RNA viruses like picornaviruses, whereas it is reported that RIG-I is responsible for type I IFN induction in response to rhabdovirus infection [34]. However, the function of Mda-5 in the innate immune response to rhabdoviridae has not yet been elucidated. Furthermore, the role of these PRRs following a RABV infection in DCs remains unknown. Therefore we wanted to determine which of the two receptors recognizes RABV. For this approach, BMDCs from Mda-5−/− mice and RIG-I−/− mice were isolated. As shown in Figure 6A, Mda-5−/− BMDCs express high levels of CD86 on their surface at 24 and 48 hpi. Of note, there is a significant reduction of CD86 surface expression on Mda-5−/− BMDCs at 12 hpi when compared to wildtype cells. Likewise, RIG-I −/− BMDCs also have a defect in BMDC activation at 12 hpi, while CD86 expression at 24 and 48 hpi is equal for RIG-I−/− and RIG-I+/+ cells (Figure 6B). In addition, it appears that while Mda-5−/− cells are able to induce type I IFN expression 12 hpi, RIG-I−/− cells have an early defect in type I IFN induction. Importantly, by 48hpi, RIG-I−/− BMDC do produce enough type I IFN to suppress VSV-GFP replication (Figure 6C). This indicates that RABV can induce BMDC activation and type I IFN via both Mda-5 and RIG-I ligation. Furthermore, any perturbation in IPS-1 mediated signaling cascades seems to affect the early response (12hpi) to RABV.

### RABV infection by itself is sufficient to induce type I IFN production, while positive feedback is necessary for sufficient BMDC activation

**Figure 7:**
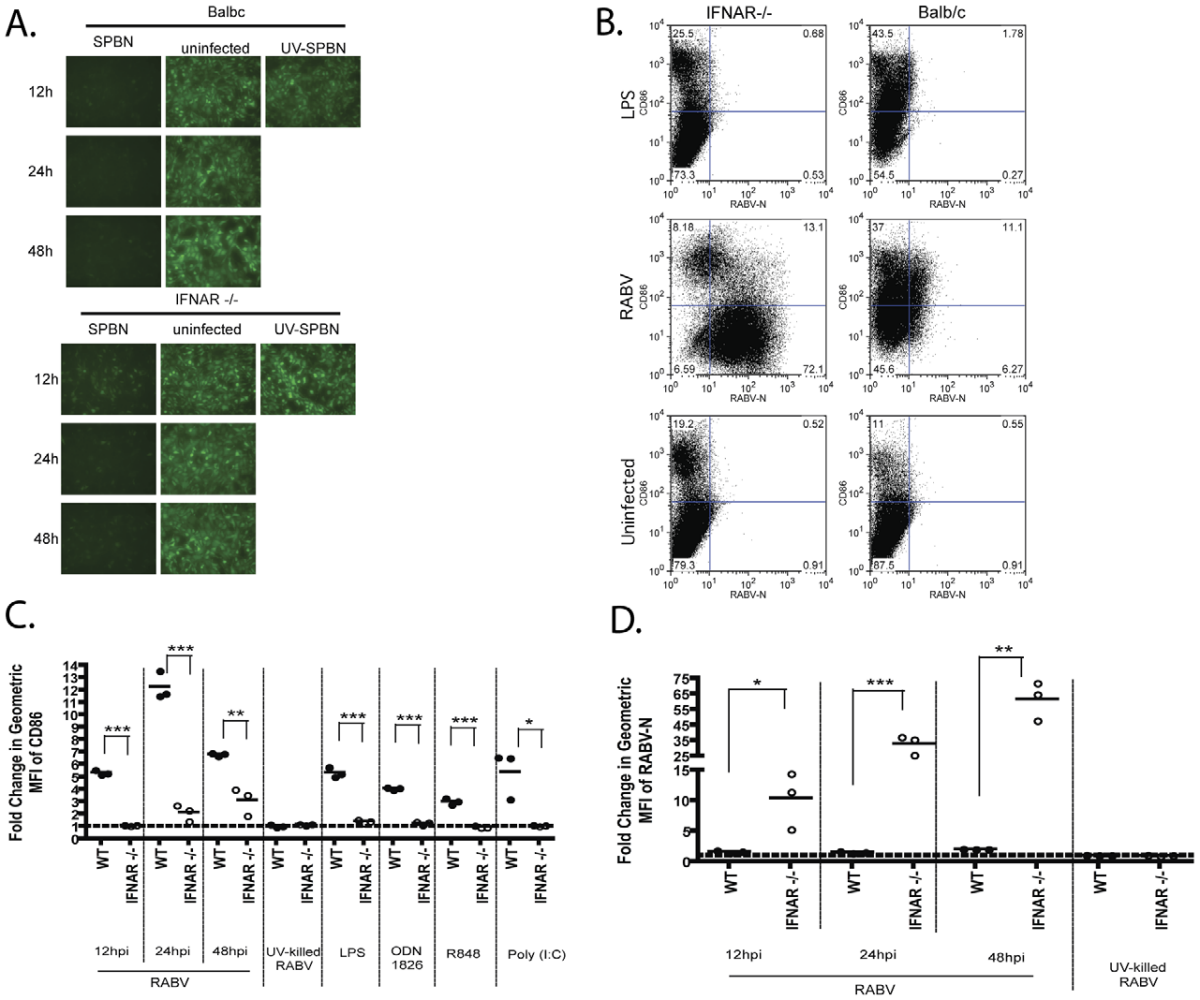
IFN signaling is critical for DC activation. (A) IFN-α/ß levels were monitored by infectivity of VSV-GFP on reporter cells. Reporter cells were pre-treated with UV-inactivated supernatant from IFNAR−/− or Balb/c BMDC. 24h after supernatant was applied, reporter cells were infected with VSV-GFP. Fluorescence indicates viral replication, and thus, lack of type I IFN. (B) One representative knock-out and wildtype mouse is shown 12hpi. (C–D) For each sample the fold increase in activation was determined by dividing the geometric mean fluorescent intensity (MFI) of the sample by the MFI of the uninfected sample. In this manner, we looked at CD86 upregulation (C) and RABV-N expression (D) following RABV infection of IFNAR−/− or Balb/c BMDC 12, 24, and 48 hours post infection. Statistical significance was determined by T-test analysis and any significance is indicated, *p<0.05, **p<0.01, ***p<0.001.

Once type I IFN is produced, it will further activate the infected cell via autocrine signaling through IFNAR. Ligation of the IFNAR initiates the Jak/STAT signaling cascade, which culminates in the upregulation of antiviral genes. In addition to antiviral genes, Jak/STAT signaling also upregulates proteins required for type I IFN induction, thus providing a positive feedback for the type I IFN pathway [2]. In order to determine how much IFN induction is directly related to RABV infection and how much is due to positive feedback that is driven by IFN-α/ß production, we infected BMDCs derived from IFNAR−/− mice, which eliminates the contribution of positive feedback. Interestingly, BMDC isolated from IFNAR−/− mice produce enough type I IFN to block VSV-GFP replication on reporter cells after 12, 24, and 48 h (Figure 7A). However, we detected a significant decrease in the CD86 cell surface expression of IFNAR−/− BMDC when compared to wt BALB/c mice (Figure 7B–C). Thus, although RABV infection is sufficient to induce type I IFN, the cells need an amplification signal in order to undergo maturation. Additionally, we see a significantly greater infection by RABV in IFNAR−/− cells, presumably due to their inability to induce antiviral gene expression (Figure 7D).

### Role of IPS-1 signaling *in vivo*

**Figure 8:**
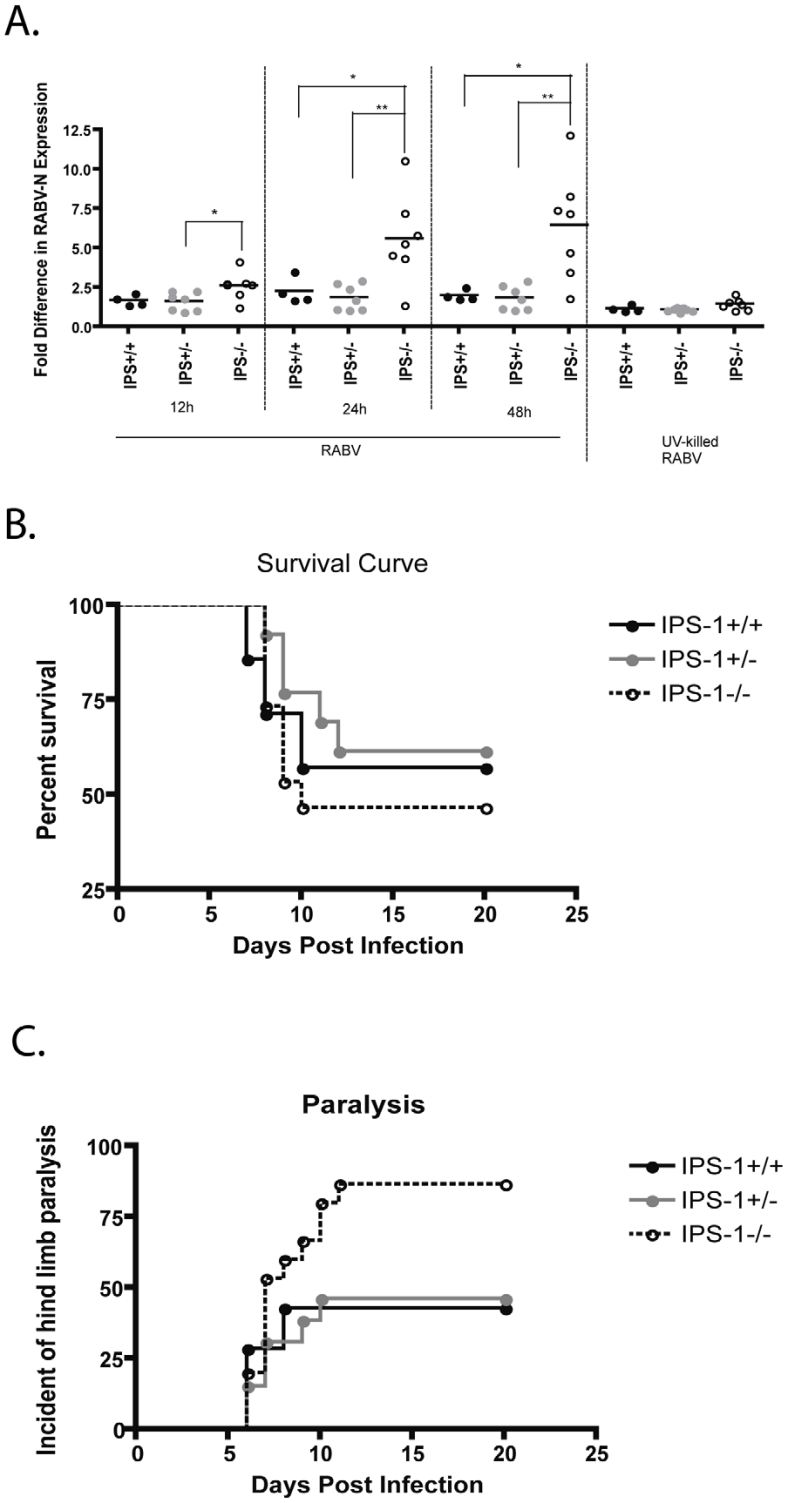
Type I IFN produced via IPS-1 signaling affects RABV infection rates and viral pathogenicity. (A) IPS-1 BMDCs deficient in type I IFN production (IPS-1−/− BMDC) are also more susceptible to RABV infection. The fold increase in RABV-N expression was determined by dividing the geometric mean fluorescent intensity (MFI) of the sample by the MFI of the uninfected sample in IPS-1 BMDCs. (B–C) In two independent experiments, IPS-1−/−, +/−, and +/+ mice were infected intramuscularly with pathogenic SPBN-N2c and their survival (B) and hind limb paralysis (C) was monitored over time. Statistical significance was determined by T-test analysis and any significance is indicated, *p<0.05, **p<0.01.

Lastly, we wanted to determine the impact that the RIG-I and Mda-5 pathways play in the *in vivo* response to RABV utilizing IPS-1 −/− mice. Interestingly, we detected that IPS-1−/− BMDC, which do not produce type I IFN, have significantly more RABV-N expression post infection (Figure 8A). This indicates that in the absence of IFN-α/ß induction, viral replication in DCs occurs at a faster rate, which should also increase viral pathogenicity. Therefore, we infected IPS-1 −/−, +/−, and +/+ mice, intramuscularly with SPBN-N2c, a recombinant RABV that is modestly pathogenic after peripheral inoculation [37]. Figure 8B shows that about 60% of the IPS-1+/+ or +/− mice lived, while only 45% of the IPS-1 −/− mice survived infection. More dramatically, nearly 90% of the IPS-1−/− mice had hind limb paralysis 11 days post infection while the IPS-1+/+ and +/− mice exhibited only about 45% paralysis (Figure 8C). This data indicates that RABV infection of IPS-1−/− is more pathogenic than RABV infection in wildtype mice.

## Discussion

It has been previously seen that RABV can infect APCs [28], [29]; however, the impact of the infection on generating an innate immune response to RABV had not been delineated. We show here that following RABV infection of APCs, unlike fibroblasts or neuronal cells, are able to produce copious amounts of type I IFN. We also determined that infected APCs do not produce novel viral progeny. A similar phenotype has also been seen following influenza infection of DCs. BMDCs become infected by the influenza strain, PR8, as seen by co-expression of influenza HA and DC marker N418 on 72% of cells. However, infected BMDC do not release viral progeny, as seen by a failure of infected DC supernatants to induce hemagglutination of chicken red blood cells [27]. Non-productive infection of DCs may have significant biological relevance over the course of an infection. Since RABV infection within APCs is easily controlled, the cells become a source of viral antigen, with little risk of spreading infection to neighboring cells. Taken together, APCs seem to be of critical importance during a RABV infection both for the prolonged production of type I IFN as well as a source of viral antigen.

In this study, we used APCs as a tool to study the PRRs used to recognize RABV following infection. Interestingly, we see that TLR-3 has no role in inducing a type I IFN response or DC activation despite its previously recognized upregulation following RABV infection [25]. However, recent publications may explain this potential discrepancy. It was reported that TLR-3 is required for the formation of Negri bodies in RABV infected cells and that these bodies are the site of viral replication [38], [39]. Furthermore, TLR-3 −/− mice are less susceptible to infection with pathogenic RABV, as seen by increased survival and lower viral titers in the brains of TLR-3 −/− animals compared to wt mice [39]. Thus, the requirement for TLR-3 by RABV may explain why it is upregulated following infection despite the fact that it is not required for a type I IFN response.

We next sought to identify whether TLR-7 was critical for DC activation and type I IFN production. To our knowledge, no one has directly examined the role of TLR-7 following a RABV infection. Of note, TLR-7 signaling does play a role in the cellular recognition of a closely related Rhabdovirus, VSV. Infection of wild type plasmacytoid DCs (pDC) with VSV induced the production of IFN-α. However, infection of pDCs from TLR7−/− or MyD88−/− mice resulted in no cytokine production [40]. Thus, indicating that single-stranded RNA derived from VSV is able to trigger TLR-7 signaling. However, in the case of RABV it appears that MyD88-dependent signaling, and thus TLR-7, is dispensable for IFN-α/ß production following infection.

On the other hand, RLR signaling via IPS-1 is critical for both the activation of DCs and production of type I IFN by infected DCs. It was shown previously that RIG-I signaling is necessary for IFN-ß promoter activity in VERO cells following recombinant RABV infection [24]. However, we show here using RIG-I−/− derived DCs that Mda-5 is also able to induce DC activation and type I IFN production. This is interesting, as Mda-5 is generally recognized as a receptor for positive stranded RNA viruses, not negative stranded RNA viruses like RABV. Of note, another negative stranded RNA virus of the Paramyxoviridae family, Sendai virus, requires MDA-5 signaling for the sustained expression of type I IFN [41].

Our data indicates that RABV can be recognized by either RIG-I or Mda-5 following infection. The use of both RIG-I and Mda-5 receptors has also been observed following infection with West Nile virus (WNV). Following WNV infection, RIG-I−/− cells had a delayed upregulation of host anti-viral genes; however, the ability to respond was conserved. Thus, indicating that another receptor was involved in recognition of WNV, this receptor was identified as Mda-5 [42], [43]. Following RABV infection in the absence of either RIG-I or Mda-5, there is a delay in the activation of BMDCs. Furthermore, RIG-I−/− BMDCs have an early defect in type I IFN production. Thus, it appears that in response to a RABV infection, both RIG-I and Mda-5 are utilized in order to rapidly induce high levels of IFN-α/ß production and DC activation.

Of note, IPS-1 +/− cells exhibited a phenotype that was intermediate to IPS-1 −/− and IPS-1 +/+ mice. This observation also supports the requirement for rapid induction of IFN-α/ß following a RABV infection. The heterozygous cells are lacking one of the IPS-1 alleles, and this may result in less functional protein in the heterozygous mice compared to homozygous wildtype mice. This again highlights the importance of a rapid response following viral infection in order to control viral replication and spread.

The type I IFN response occurs in two phases after infection: the induction of IFN-α/ß following recognition of the pathogen by a PRR and then autocrine or paracrine signaling by IFN-α/ß through the IFNAR to induce upregulation of many other genes. Included among the genes that are upregulated in response to IFNAR signaling are several genes required for PRR signal transduction [2]. In this manner, the infected cell undergoes positive feedback to increase both the host response and PRR signaling. We wanted to identify which arm of the IFN response was responsible for the effects we observed following RABV infection of DCs, viral induction or IFN-α/ß amplification. We saw that both wt and IFNAR −/− mice are able to induce type I IFN production, thus highlighting the host's ability to rapidly induce IFN-α/ß following infection with RABV and indicating that the amplification of IPS-1 signaling by IFNAR signaling is not a critical factor in the induction of type I IFN. Surprisingly, we see that in the absence of IFNAR signaling, there is very little BMDC activation. Thus, it appears that DC activation occurs via IFN-α/ß signaling and is not a direct consequence of viral infection. This fact highlights the importance of a type I IFN response in initiating the adaptive immune response following infection with RABV.

Lastly, we sought to determine the biological relevance of IPS-1 mediated PRR signaling following infection with a pathogenic strain of RABV. Although this experiment did not focus specifically on type I IFN production by DCs, it indicates how IPS-1 signaling, and thus IFN-α/ß production and DC activation, impacts the prognosis of infected animals. We saw that 87% of the IPS-1−/− mice in the study became paralyzed, whereas only about 45% of the IPS-1 +/+ or +/− mice exhibited signs of paralysis. There is some data that suggests paralysis following a RABV infection is an early symptom of disease. In humans who present with the less common paralytic rabies, their survival time is slightly longer [44]. Although not significant, this data supports the fact that an early, rapid type I IFN response is an important factor mediating RABV disease outcome. Of note, opposed to vaccine strain of RABV used in the BMDC experiments, the pathogenic RABV strain, SPBN-N2c, infects mostly neurons [37] and we showed here that RABV is able to suppress the type I IFN response in neurons by 12hpi (Figure 1). Despite this limitation, there is no other model to study RABV pathogenicity. The role that antigen presenting cells play in initiating the immune response to RABV *in vivo* should also be investigated further. It is known that pathogenic RABV is less immunogenic than vaccine strains of RABV [45] thus it is likely that pathogenic RABV avoids or alters infection of DC in order to elicit a lesser immune response.

In summary, we show here that RABV replication is cell type dependent; namely, RABV is able to antagonize the induction of type I IFN in fibroblast and neuronal cells but is unable to inhibit IFN-α/ß induction in APCs. Furthermore, in APCs RABV infection is non-productive due to a defect in viral transcription, and no viral production is observed. Infection of BMDCs allowed us to delineate that RABV is exclusively recognized by either RIG-I or Mda-5 and both receptors are required for a rapid type I IFN response to RABV. This finding has significant implications for the development of a RABV-based vaccine vector. In light of these results, a recombinant RABV expressing a TLR agonist may allow for RABV recognition via TLRs. Such a response may potentiate the type I IFN response and induce better protection in a vaccine setting. We also show here that BMDC activation is secondary to IFN-α/ß induction and requires IFNAR. In addition, IPS-1 mediated signaling does have a role *in vivo*, as it seems to play a critical role in preventing RABV pathogenesis following RABV challenge.

## Materials and Methods

### Ethics statement

All animals were handled in strict accordance with good animal practice as defined by the relevant international (Association for Assessment and Accreditation of Laboratory Animal Care (AAALAC) (Accreditation Status TJU: Full)) and national (TJU Animal Welfare Assurance Number: A3085-01), and all animal work was approved by the Institutional Animal Care and Use Committee (IACUC) at Thomas Jefferson University TJU. Animal use protocols are written and approved in accordance with Public Health Service Policy on Humane Care and Use of Laboratory Animals, The Guide for the Care and Use of Laboratory Animals. TJU IACUC protocol number 414A, 414G, and 414I were utilized in this study.

### Cell lines

The fibroblast cell line used in these studies is a cell clone of BHK-21 (ATCC: CCL-10), BSR. The neuronal cell line used in these studies is a neuroblastoma cell line referred to as NA [46]. The antigen presenting cell lines used here were JAWSII (ATCC: CRL-11904) and Raw264.7 (ATCC: TIB-71).

### Mice

Mice used in this work are as follows: B6/129S1-Tlr3tm1Flv/J (TLR-3 −/−, Jackson Laboratory, stock 005217); B6129SF1/J (Jackson Laboratory, stock 101043); MyD88−/− [47]; C57BL/6 (NIH); IPS-1 [48]; Mda-5 −/− [33]; RIG-I [49]; BALB/c (NIH); and IFNAR−/− mice [50].

For pathogenicity study, IPS-1 mice were genotyped as described previously [48]. IPS-1 +/+ (n = 7), IPS-1 +/− (n = 13), and IPS-1 −/− (n = 15) mice were infected intramuscularly with 10^6^ ffu SBPN-N2c [37]. The weight of the mice was monitored daily, and the animals were euthanized after losing 25% of their body weight, which indicates a severe rabies infection.

### IFN sensitivity assay

Cellular supernatants were assessed for the ability to inhibit vesicular stomatitis virus (VSV) replication as described previously [4]. Briefly, the cell line of interest was infected with the vaccine strain of RABV, SPBN, at a multiplicity of infection (MOI) of 10, and supernatant was collected at various time points post infection. Alternatively, supernatant from infected BMDCs was used. The supernatants were UV-deactivated with a 254nm UV light source for 15 min. UV-deactivated viral supernatant was then diluted 1∶10 in RPMI-1640 and added to a reporter cell line (either NA, for cell line experiments or 3T3 cells, for BMDC experiements). Following the 24 h pre-treatment, reporter cells were infected with VSV- expressing GFP at a MOI of 5 for 5–8 h. VSV replication was determined by fluorescence under a UV light source.

### ELISA assays

For IFN-ß ELISA (PML Laboratories) the manufacturer's protocol was followed with the following modification: 50 µl of sample or standard was loaded into the 96-well plate. For IL-6 ELISA (eBioscience) the manufacturer's protocol was followed. Briefly, 5 µg/ml coating antibody was added to MaxiSorb (Nunc) plates and kept at 4°C over night. Wells were then washed with 0.05% Tween-20/PBS and blocked with Assay Buffer (eBioscience) for 2 hours. Plates were again washed with 0.05% Tween-20/PBS and then 100 µl standard or sample and 50 µl Biotin-Conjugate was added to the plate. Plates were incubated at room temperature for 2 hours, on a microplate shaker set at 200 rpm, and then washed with 0.05% Tween-20/PBS. Subsequently, wells were incubated with Streptavidin-HRP at room temperature for 1 hour, on a microplate shaker set at 200 rpm. The wells were washed and developed with 100 µl of Substrate Solution for 10 min followed by the addition of 100 µl of Stop Solution. Absorbance at 450 nm was recorded for each well. For both ELISAs a fourth-order non-linear regression curve (Prism software, GraphPad version 4.00) was fit to the standard curve and used to determine the concentration of the unknown samples.

### One-step growth curve

BSR, NA, JAWSII and Raw264.7 cells were infected with SPBN at a MOI of 10. Following 60 min incubation at 37°C, the virus was aspirated, and cells were washed twice with PBS to remove any virus that had not yet infected the cells. Media was then added to the cells, and, at indicated time points, 0.3ml of supernatant was removed and stored at 4°C. The aliquots were titered in duplicate on BSR cells.

### Quantitative real-time PCR

Messenger and genomic RABV-N RNA in SPBN (MOI-10) infected BSR and JAWSII cells was determined by TaqMan probe-based real-time PCR as described previously [4], [37].

### Western blotting

Western blotting was performed as described previously [51].

### Bone marrow derived DC (BMDC) differentiation and infection

BMDCs were differentiated as described previously [52]. Briefly, bone marrow (BM) was obtained from the mouse's tibia and femur. Following red blood cell lysis using ACK lysis buffer (Invitrogen), the BM cells were cultured in 24-well costar plates at a density of 1 million cells per ml in the presence of 10ng/ml GM-CSF (Peprotech). During the 7 day culture, the cells were washed once by aspirating 600µl of media from the wells and adding 1ml of fresh media supplemented with 10ng/ml GM-CSF. On the seventh day of culture, the non-adherent and semi-adherent cells were collected and used as the BMDCs population.

BMDCs were plated in 12-well plates (Nunc) at a maximum density of 1 million cells per ml of media. BMDCs were infected with SPBN at an MOI of 10 or ΔNS1/PR8 [53] at an MOI of 1. SPBN was harvested from BSR cells grown in serum free Opti-Pro media 4 and 7 dpi. Viral supernatant was pooled and spun at 1600 rpm for 10 min to remove cell debris. Alternatively, cells were left uninfected or stimulated with UV-deactivated SPBN, LPS (2µg/ml, Sigma), ODN1826 (2µM, InvivoGen), R848 (1 µg/ml, InvivoGen), or poly(I:C) (50 µg/ml, InvivoGen). Infected BMDC were kept at 37°C with 5% CO_2_ for 12, 24, or 48 h.

### Flow cytometry

Following differentiation of BMDC, cells were characterized for expression of DC markers. Briefly, cells were washed in FACS buffer (2% BSA/PBS) and blocked at 4°C for 30–60 m with 2µl rat anti-mouse CD16/CD32 (Fc block) (BD Biosciences Pharmigen) in 100µl FACS. Cells were then stained with APC-CD11b, PerCP-B220, and FITC-CD11c (BD Biosciences Pharmingen) for 30 min at RT. After staining, cells were washed with FACS buffer and fixed with Cytofix (BD Biosciences) for 16–18 hours at 4°C. Samples were washed and resuspended in 300 µl of FACS buffer. Samples were analyzed on BD FACS Calibur and a minimum of 50,000 events were counted.

Following infection, BMDCs were analyzed for the expression of activation markers. At each given timepoint, BMDCs were removed from wells with cell scrappers and spun at 1600rpm for 5 min. Cells were then blocked at 4°C for 30–60 min with Fc block in 100µl FACS. Cells were then stained with APC-CD11c and PE-CD86 (BD Biosciences Pharmingen) for 30 min at RT. After staining, cells were washed with FACS buffer and fixed with Cytofix (BD Biosciences) for 16–18 hours at 4°C. Cells were then washed twice in Perm/Wash Buffer (BD Bioscience) and then stained with FITC-anti RABV-N (Centacor, Inc) for 30 min at RT. After staining, cells were washed with Perm/Wash buffer and then resuspended in 300 µl of FACS buffer. Samples were analyzed on BD FACS Calibur and 20,000–30,000 APC^+^ events were counted.

### Statistical analysis

All data were analyzed by Prism software (GraphPad, version 4.00). To compare two groups of data we used an un-paired, two-tailed T-test. For all tests, the following notations are used to indicate significance between two groups: *p<0.05, **p<0.01, ***p<0.001.
